# Leaf and Canopy Traits Associated with Stay-Green Expression Are Closely Related to Yield Components of Wheat Genotypes with Contrasting Tolerance to Water Stress

**DOI:** 10.3390/plants11030292

**Published:** 2022-01-22

**Authors:** Felipe Moraga, Marta Alcaíno, Iván Matus, Dalma Castillo, Alejandro del Pozo

**Affiliations:** 1 Centro de Mejoramiento Genético y Fenómica Vegetal, Facultad de Ciencias Agrarias, Universidad de Talca, Talca 3460000, Chile; moraga.fe@gmail.com (F.M.); malcaino10@alumnos.utalca.cl (M.A.); 2 Centro de Biotecnología Vegetal, Facultad de Ciencias de la Vida, Universidad Andrés Bello, Santiago 8370146, Chile; 3 CRI-Quilamapu, Instituto de Investigaciones Agropecuarias, Chillán 3800062, Chile; imatus@inia.cl (I.M.); dalma.castillo@inia.cl (D.C.)

**Keywords:** agronomical traits, chlorophyll content, NDVI, stay-green trait

## Abstract

The onset and rate of senescence influence key agronomical traits, including grain yield (GY). Our objective was to assess the relationships between stay-green and GY in a set of fourteen spring bread wheat (*Triticum aestivum* L.) genotypes with contrasting tolerance to water stress. Based on leaf chlorophyll content index (Chl) and normalized vegetation index (NDVI) measurements, the senescence dynamics at leaf and canopy levels, respectively, were quantified. Parameters describing the dynamics of senescence were examined in glasshouse and field experiments under well-watered (WW) and water-limited (WL) regimes, and they included the following stay-green traits: maximum NDVI or Chl near to anthesis (*NDVI*_max_, *Chl*_max_), the senescence rate (*SR*, *rate*), the area under curve (*Area*_NDVI_, *Area*_Chl_), and the time from anthesis to 10 (*t*_onset_), 50 (*t*_50_, *X*_50_) and 90% (*t*_90_) senescence. Our results revealed that specific stay-green traits were significantly different among genotypes and water regimes in both glasshouse and field experiments. GY was positively correlated with *t*_total_ (0.42), *t*_onset_ (0.62) and *NDVI*_dif_ (0.63). Under WL, *NDVI*_dif_ and *NDVI*_max_ correlated with GY (0.66–0.58), but only *t*_50_ correlated with GY under WW (0.62), indicating that phenotyping of stay-green trait is a useful tool for tracking the dynamics of senescence in WW and WL environments.

## 1. Introduction

Water deficit is the major environmental factor that reduces growth, quality and productivity in the world’s most important cultivated crops [1]. Depending on its intensity, frequency, and combination with different stress factors, the grain yield (GY) can be reduced by more than 50%, and this problem is expected to increase with the projected global climate change [2,3].

In Mediterranean-climate areas, wheat is often exposed to a progressive water deficit from flowering to the grain filling stage, leading to terminal drought stress [4,5]. Therefore, understanding the mechanisms underlying plant tolerance to drought scenarios is essential to enhance crop resilience, considering the impact of terminal drought on crop productivity in rain-fed areas of Mediterranean environments.

Senescence is the last developmental stage of plant cells, tissues, and organs and, in the case of monocarpic species such as wheat, leaf senescence occurs along with the whole plant. In annual crops, leaf senescence is under the control of a highly regulated genetic program, which ensures the remobilization and efficient translocation of assimilates such as carbohydrates, amino acids, and mineral nutrients accumulated in vegetative tissues towards developing grains [6,7]. Therefore, the availability of these assimilates in the grain is strongly influenced by the senescence timing. Consequently, the onset and rate of senescence influence key agronomical traits, including grain yield and quality, as well as nutrient content [8,9,10].

Leaf senescence usually starts from the tip or margins of a leaf towards its base with the progressive dismantling of mesophyll cells in a coordinated manner to remobilize nutrients and ensure reproductive success. Chloroplasts are the first organelles to be dismantled, allowing a major portion of leaf lipid and proteins to be recycled, while the mitochondria and nucleus remain intact until the final stages of senescence [6,11], often associated with the visual loss of chlorophyll, which corresponds to the first external symptoms of leaf senescence. Nonetheless, the senescence process also involves a series of structural, metabolic and transcriptional changes [6,12].

In wheat, delayed senescence or stay-green phenotypes often correlate with yield due to extended periods of photosynthesis and the maintenance of the green canopy [13,14], although this is not always the case [8,15]. Specifically, stay-green refers to a heritable delayed foliar senescence character, which may improve the grain filling process even under stress conditions [14]. Indeed, upon exposure to adverse environmental conditions, such as biotic and abiotic stress, the plant can initiate the process of senescence as an adaptive response to promote survival and reproduction [11,16]. In this context, drought prematurely induces the process of senescence, which leads to a loss in chlorophyll content, a decrease in canopy size and activity of photosynthesis, and reduced grain yield [17]. Therefore, the relationship between senescence (i.e., senescence or stay-green traits) and plant productivity is complex, and its positive effect depends on the plant species, genotype, productivity parameter measured (i.e., biomass, grain yield), and environment [8,14,18].

Different methods have previously been used to quantify the dynamics of senescence in the field at leaf and whole-plant levels. For instance, rapid evaluation by visual observation has made it possible to describe the fractional loss in the green leaf area of wheat [19,20], but the rate of chlorophyll loss taken for an individual leaf with the Minolta SPAD meter has also been reported to be useful [13,21]. Meanwhile, high-throughput phenotyping based on the normalized difference vegetative index (NDVI) has been used successfully to quantify the dynamics of senescence (i.e., stay-green traits), and might be useful in plant breeding [22,23,24,25]. As far we are aware, direct comparisons between leaf and canopy approaches to assess the stay-green dynamic have not been performed yet. Indeed, most of the studies that have performed comparisons between leaf and canopy indexes focus on estimating the crop N status for improving N management through dose optimization [26,27]. For other crops, it has been established that these properties also affect the expected yield [28].

This study aimed to assess the association between the stay-green and agronomical traits of fourteen spring bread wheat (*Triticum aestivum* L.) genotypes with contrasting tolerance to water stress. The set of genotypes was selected from a collection of 384 cultivars and advanced semi-dwarf lines of spring bread wheat characterized for different physiological and agronomical traits in field conditions [5], and evaluated in field and greenhouse trials, under water-limited (WL) and well-watered (WW) regimes. The stay-green dynamic was evaluated at leaf and whole-plant levels based on leaf chlorophyll content (measured with a portable chlorophyll meter) and NDVI measurements, respectively. The adjustment of the chlorophyll content and NDVI data made it possible to obtain information about the senescence dynamic (stay-green traits) between genotypes and is useful as an indicator of genotypic performance under conditions with different levels of water stress and can be used in the identification and selection of superior wheat genotypes through the breeding process.

## 2. Results

### 2.1. Dynamic of Flag Leaf Senescence

**Figure 1:**
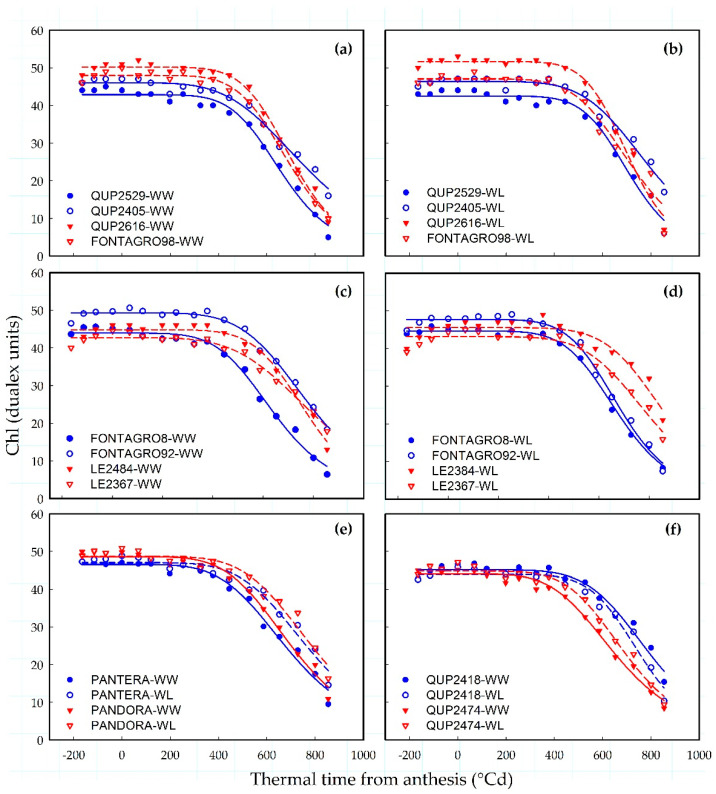
Dynamics of flag leaf senescence in a set of contrasting spring bread wheat genotypes from glasshouse experiment. (**a**–**d**) Variation among lines grown under (**a**–**c**) well-watered (WW) and (**b**–**d**) water-limited (WL) conditions. (**e**,**f**) Variation between water regimes. In (**a**–**f**), each point represents the average of four replicates but curves were fitted using individual replicates. Symbols represent measured experimental chlorophyll content index (Chl) values, whereas the lines represent the regression curves fitted with Equation (1).

**Figure 2:**
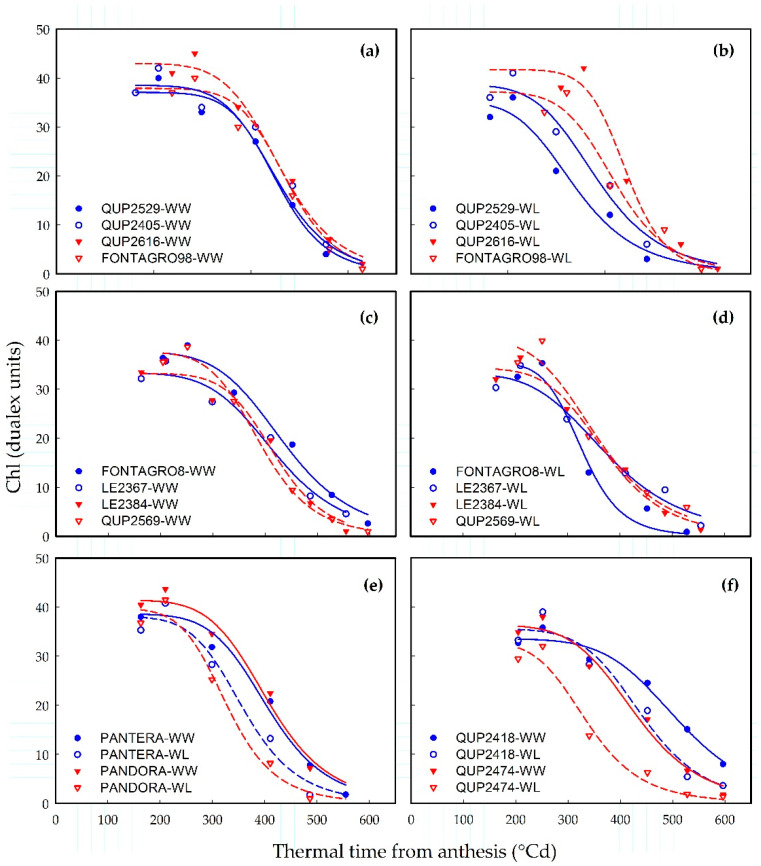
Dynamics of flag leaf senescence in a set of contrasting spring bread wheat genotypes from field experiment. (**a**–**d**) Panels highlight the variation among lines grown under (**a**–**c**) well-watered (WW) and (**b**–**d**) water-limited (WL) conditions. (**e**,**f**) Panels highlight the variation between water regimes. In (**a**–**f**) each point represent the average of three replicates but curves were fitted using individual replicates. Symbols represent measured experimental chlorophyll content index (Chl) values, whereas the lines represent the regression curves fitted with Equation (1).

The dynamics of flag leaf chlorophyll content (Chl) followed a logistic function (Equation (1)) against accumulated thermal time after anthesis, in both glasshouse and field experiments (Figure 1 and Figure 2).

#### 2.1.1. Glasshouse Experiment

The coefficients of determination (R^2^) of the regression model were greater than 0.85 for all the pots. Significant differences were observed for leaf stay-green traits among genotypes and between water regimes (Table 1), except for the maximum chlorophyll accumulated (*Chl*_max_), the time from anthesis to loss of 50% of the leaf chlorophyll content (*t*_50_), and area under Chl curve (*Area*_chl_), in which the effect of water regime was not significant. The genotype × water regime (G × W) interaction was not significant (Table 1).

Under a water-limited (WL) regime, the genotypes showed a delayed onset of senescence (*t*_onset_) and a significant increase (*p* < 0.01) in the senescence rate (*rate*), while the duration of the persistence phase (*decay*) and time to reach 90% of senescence (*t*_total_) decreased significantly (*p* < 0.05, Table 1). Some examples of the dynamics of chlorophyll loss between genotypes evaluated under WL and well-watered (WW) regimes are shown in Figure 1. The genotype QUP2616 featured the highest *Chl*_max_ (50 and 52 dualex units under WW and WL, respectively) in contrast to QUP2529 (43 dualex units) under both water regimes (Figure 1a,b and Table 1). Furthermore, the thermal time to reach 50% senescence (*t*_50_) were highest in the genotypes LE 2367 (WW: 818 °Cd) and LE 2384 (WL: 824 °Cd) than FONTAGRO8 (Table 1), which showed the lowest values under both water conditions (WW: 638 °Cd and WL: 682 °Cd; Figure 1c,d). Compared to FONTAGRO8, the genotype LE 2384 had a delayed onset of senescence (*t*_onset_) under both water regimes (Figure 1c,d and Table 1). Under the WL regime, the *t*_total_ and *decay* were higher in QUP2405 than the genotypes FONTAGRO98 and QUP2616 (Figure 1b and Table 1). Meanwhile, PANTERA-INIA and PANDORA-INIA exhibited a similar pattern of leaf senescence (Figure 1e). Compared to QUP2418, the genotype QUP2474 exhibited an accelerated onset of senescence (lowest *t*_onset_) under both water conditions (Figure 1f).

#### 2.1.2. Field Experiment

The Chl data fitted well to Equation (1) and the coefficient of determination ranged from 0.91 to 0.99 (Figure 2). The water regime and genotypic effects were significant (*p* < 0.05) for *Area*_chl_, *t*_50_, and *t*_total_ (Table 1). Furthermore, *Chl*_max_ was significantly different among genotypes. For all the leaf senescence traits, the G × E interaction was not significant (Table 1).

In response to WL, the leaf senescence trait *t*_onset_ was the most sensitive to changes in water regime, but *t*_50_, *t*_total_ and *Area*_chl_ also decreased significantly (*p* < 0.001; Table 1). The maximum chlorophyll content (*Chl*_max_) was highest for genotype QUP2616 (43 and 42 dualex units under WW and WL, respectively), whereas LE 2367 showed the lowest values of *Chl*_max_ (33 dualex units) under both water regimes (Figure 2a–d and Table 1). Under the WL regime, the genotype FONTAGRO8 showed an accelerated pattern of senescence (Figure 2d) and was the earliest genotype to loss 50% of *Chl*_max_ at 336 °Cd from anthesis (Table 1). However, QUP2569 featured the lowest *t*_50_ under WW regime (392 °Cd; Figure 2c and Table 1). Meanwhile, PANTERA-INIA and PANDORA-INIA showed a similar pattern in the dynamic of leaf senescence under each regime (Figure 2e). Compared to QUP2418, QUP2474 rapidly lost 90% of Chl (lower *t*_total_) under WW and WL at 587 and 494 °Cd from anthesis, respectively (Table 1). The senescence pattern of QUP2418 in response to WL was similar to that observed in QUP2474 under WW (Figure 2f).

### 2.2. Dynamic of Canopy Senescence

#### Field Experiment

**Figure 3:**
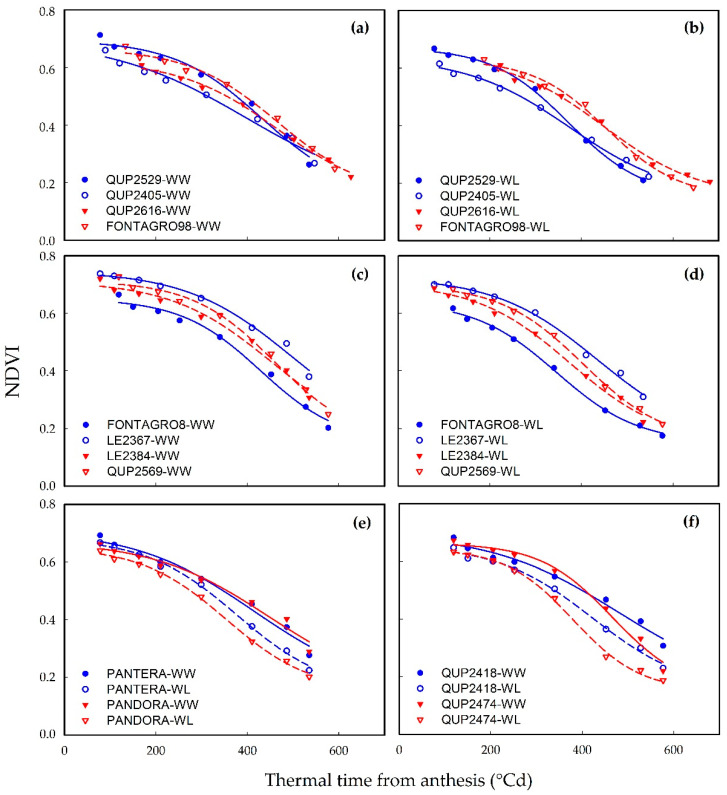
Dynamic of canopy senescence in a set of contrasting spring bread wheat genotypes from field experiment. (**a**–**d**) Panels highlight the variation among lines grown under (**a**–**c**) well-watered (WW) and (**b**–**d**) water-limited (WL) conditions. (**e**,**f**) Panels highlight the variation between water regimes. In (**a**–**f**), each point represents the average of four replicates but the curves were fitted using individual replicates. Symbols represent measured experimental normalized difference vegetative index (NDVI) values, whereas the lines represent the regression curves fitted with the Equation (2).

Equation (2) provided a close fit (0.94 ≤ R^2^ ≤ 0.99) to the NDVI data and captured the effects of genotype and treatment (Figure 3). Significant differences (*p* < 0.001) in the dynamics of NDVI were observed between WW and WL treatment and among the genotypes for all stay-green traits (Table 2). The G × E interaction was not significant, except for the senescence rate (*SR*). In response to WL, the leaf senescence traits *NDVI*_dif_, *NDVI*_max_, *Area*_NDVI_, and *X*_50_ decreased, but *SR* increased significantly (*p* < 0.001; Table 2).

Some examples of the dynamics of canopy senescence between genotypes are shown in Figure 3. QUP2405 was the earliest genotype to lose 50% *NDVI*_max_ under WW conditions at 391 °Cd from anthesis but also showed the lowest *SR* under both WW and WL regime (Figure 3a,b and Table 2). In response to WL, the senescence rate increased in most genotypes, except for genotypes LE 2367, LE 2384, QUP2616, QUP2569, and QUP2529, which maintained a similar *SR* under both water regimes (Table 2). The genotype LE 2367 showed the highest *NDVI*_max_ and *Area*_NDVI_ under both water regimes, while FONTAGRO 8 featured the lowest *Area*_NDVI_ and the genotypes QUP2616 and FONTAGRO 98 showed the lowest *NDVI*_max_ under WW and WL conditions, respectively (Figure 3a–d and Table 2). Furthermore, FONTAGRO 8 showed accelerated senescence (lowest *SR*) and was the earliest genotype to loss 50% of *NDVI*_max_ (*X*_50_; at 345 °Cd from anthesis) under WL regime (Figure 3d and Table 2). Meanwhile, PANTERA-INIA and PANDORA-INIA displayed a similar pattern of the dynamic of canopy senescence under both water conditions (Figure 3e). Compared to QUP2418, the genotype QUP2474 showed a decrease in *NDVI*_max_, *X*_50_, and *Area*_NDVI_, and a higher rate of senescence under both water regimes (Figure 3f and Table 2).

### 2.3. Agronomical Traits and Relationships with Stay-Green Traits

#### 2.3.1. Glasshouse Experiment

The grain yield (GY), kernels per spike (KS) and thousand-kernel weight (TKW) were significantly different among genotypes and between water regimes, but the harvest index (HI) was only different among genotypes (Table 3). However, G × E interaction was not significant for any agronomical traits.

Under WW, the average GY in the fourteen wheat genotypes was 17 g plant^−1^ but some genotypes produced up to 20 g plant^−1^ such as QUP2418, which also featured the highest TKW, about 60 g. Under WL, GY was significantly reduced, by 23% (*p* < 0.001; Table 3), compared to the WW regime. Furthermore, KS slightly decreased in response to the WL regime, but TKW increased significantly (*p* < 0.001; Table 3). FONTAGRO92, LE 2367, and LE 2384 were the genotypes with the highest yielding under WL, and QUP2546 featured the lowest GY and HI under both water regimes (Table 3). Compared to WW, PANTERA-INIA was the genotype that least reduced GY (12%) in response to WL, but QUP2418 was the most sensitive, decreasing GY by 32%.

**Figure 4:**
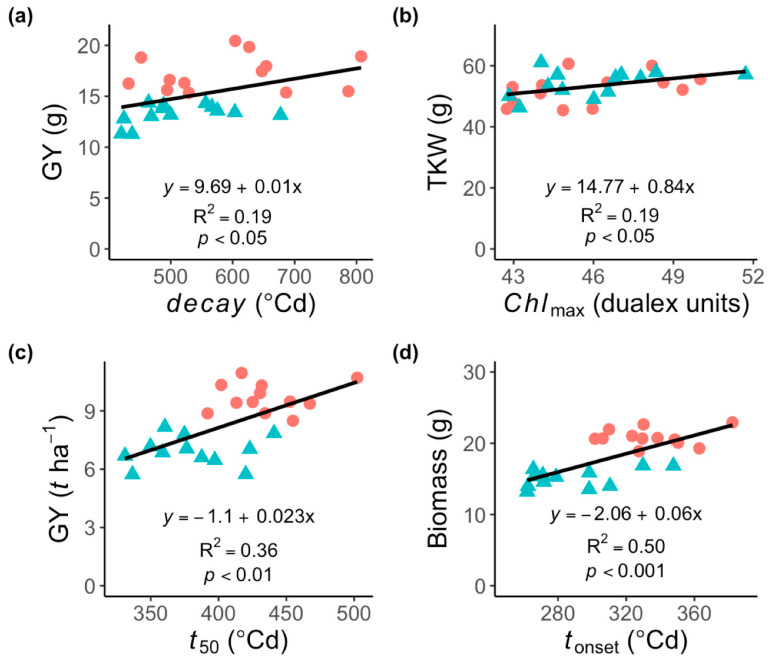
Relationship between agronomical and leaf senescence traits associated with stay-green expression in fourteen spring bread wheat genotypes. (**a**) Association between grain yield (GY) and *decay*, and (**b**) between thousand-kernel weight (TKW) and *Chl*_max_ in the glasshouse experiment. (**c**) Association between grain yield (GY) and *t*_50_ and (**d**) between biomass and *t*_90_ in the field experiment. Closed symbols represent the well-watered (**circles**) and water-limited (**triangles**) regimes.

An analysis including both water regimes revealed that GY and KS were significantly (r = 0.42–0.44, *p* < 0.05) correlated with some stay-green traits estimated at the leaf level, including *t*_total_ and *decay* (Table 4). Furthermore, TKW was positively correlated (r = 0.44, *p* < 0.05) with *Chl_max_* (Figure 4b), but no significant correlations were found between HI and stay-green traits (Table 4).

#### 2.3.2. Field Experiment

The GY, KS and spikes per square meter (SM2) were significantly different among genotypes and between water regimes, but TKW was only different among genotypes (Table 5). The G × E interaction was significant (*p* < 0.05; Table 5) for GY, KS and TKW. Compared to WW, GY was significantly (*p* < 0.001) reduced by 29% in response to WL. Under WW, the average GY of the fourteen wheat genotypes was 9.5 *t* ha^−1^. The highest GY under WW was for LE 2384 (10.9 *t* ha^−1^) and QUP2418 (10.7 *t* ha^−1^), but they also showed the best performance under WL with values of 7.8 *t* ha^−1^ for both genotypes (Table 5). FONTAGRO 92 recorded the lowest GY with values of 7.9 and 5.2 *t* ha^−1^ in WW and WL conditions, respectively. Compared to WW, PANTERA-INIA was the genotype that least reduced GY (21%) in response to WL but FONTAGRO 8 was the most sensitive, decreasing GY by 44% (Table 5).

An analysis including both water regimes showed that GY, biomass, and SM2 were positively correlated (r = 0.44–0.72, *p* < 0.05) with leaf senescence traits, including *t*_onset_, *t*_50_, *t*_total_ (Table 4 and Figure 4c,d). Furthermore, the area under Chl curve was positively correlated with GY and biomass, but no significant correlations were found between HI and leaf senescence traits. However, Chl content values recorded at grain filling (Chlgf) were positive correlated with GY (r = 0.71, *p* < 0.001), biomass (r = 0.66, *p* < 0.001) and SM2 (r = 0.63, *p* < 0.01), but negatively with KS (r = −0.53, *p* < 0.01; Table 4).

**Figure 5:**
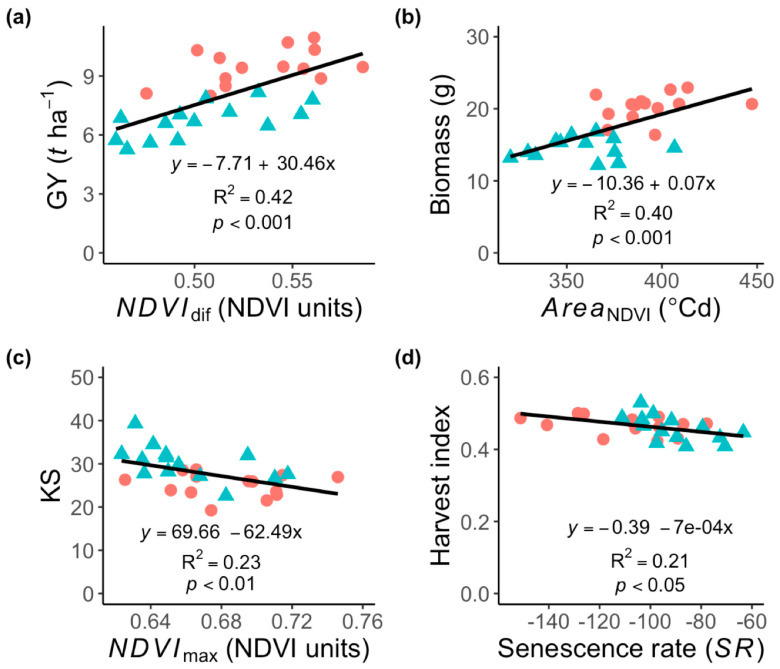
Relationship between agronomical and canopy senescence traits associated to stay-green expression in fourteen spring bread wheat genotypes growth under field condition. (**a**) Association between grain yield (GY) and *NDVI*_dif_, (**b**) biomass and *Area*_NDVI_, (**c**) kernels per spike (KS) and *NDVI*_max_ and (**d**) harvest index and senescence rate (*SR*) in the field experiment. Closed symbols represent the well-watered (**circles**) and water-limited (**triangles**) irrigation.

In addition, several canopy senescence traits were positively correlated (r = 0.42–0.65, *p* < 0.05) with GY, biomass and SM2 (Table 4), but more strongly with *NDVI*_dif_ and Area under NDVI curve (Figure 5). By contrast, *SR* exhibited a negative and significant association (r = 0.42–0.65, *p* < 0.05) with GY and biomass. KS was positively associated with *SR*, but negatively with *NDVI*_dif_ and *NDVI*_max_ (Table 4). Besides, HI was positively correlated with *NDVI*_dif_, but negatively with *SR*. Moreover, the NDVI values recorded at grain filling (NDVIgf) were strongly correlated with GY (r = 0.84, *p* < 0.001), biomass (r = 0.83, *p* < 0.001), and SM2 (r = 0.76, *p* < 0.001), but negatively with KS (r = −0.66, *p* < 0.001; Table 4).

Under WL, the canopy senescence traits *NDVI*_dif_ and *NDVI*_max_ were correlated with GY (0.66-0.58), KS (−0.57, −0.54), and SM2 (0.58–0.55), but also the *SR* was negatively correlated with HI (−0.59; Supplementary Materials Table S1). By contrast, only the leaf senescence trait *rate* was positively correlated with KS (0.60). However, NDVIgf was strongly correlated with almost agronomical traits, with the exception of TKW and HI. Under WW, *X*_50_ and NDVIgf were positively correlated with KS and SM2, respectively (0.65, 0.55; Supplementary Materials Table S1). Furthermore, Biomass was negatively correlated with *Chl*_max_ (−0.63) and Chla (−0.62).

## 3. Discussion

### 3.1. Effects of Water Stress on the Senescence Dynamics

In rainfed environments, wheat is often exposed to a progressive water deficit, mostly near to anthesis and during grain filling, leading to terminal drought stress [29]. In response to drought, senescence can be prematurely induced, which impacts crop growth and development, and consequently affects yield and productivity [8].

The results presented here indicated that leaf and canopy senescence traits associated with stay-green expression were significantly different among wheat genotypes and water regimes (Table 1 and Table 2). In response to the water-limited (WL) regime, the senescence timing was affected and, consequently, a rapid leaf chlorophyll loss (*decay*) and high senescence rate were reported in the glasshouse experiment (Table 1). Compared to the well-watered (WW) regime, the senescence rate estimated at leaf level was accelerated under glasshouse conditions. The susceptible genotypes LE 2384, FONTAGRO 98, QUP2616, and QUP2569 growth under glasshouse conditions featured a shorter chlorophyll loss phase (*decay*), and higher senescence rate in response to WL (Table 1). By contrast, the tolerant genotypes LE 2367, QUP2405, QUP2574, and PANTERA-INIA exhibited a longer duration of chlorophyll loss (*decay*) and total leaf senescence (*t*_total_) (Table 1).

As we expected, the effect of water regime on leaf senescence traits in the field experiment was different from glasshouse conditions, and except for FONTAGRO 8 and QUP2474, a decreased senescence rate was observed among genotypes (Table 1). In response to WL stress, a shorter persistence phase and accelerated time to the onset (*t*_onset_), midpoint (*t*_50_), and total (*t*_total_) leaf senescence were reported under field conditions. While the genotypes QUP2616, QUP2569, QUP2418, LE 2367, and FONTAGRO 98 exhibited a longer duration of chlorophyll loss (*decay*), the tolerant genotypes FONTAGRO 8, PANTERA-INIA, and QUP2474 exhibited shorter and fast leaf senescence (Table 1).

The present study showed that phenotypic measurements of chlorophyll content provide information about stay-green dynamics among genotypes and capture the effects of different water regimes (Figure 1 and Figure 2 and Table 1). This is in agreement with previous results from different cereals, including sorghum [30], maize [31], and wheat [13]. Meanwhile, non-destructive phenotyping based on the use of proximal sensing to obtain the vegetative index, such as NDVI, has been used successfully to track senescence dynamics in wheat [23,25,32] and oats [33] and could be useful in crop breeding. The results showed that the sigmoid model of NDVI data obtained with the proximal sensor Greenseeker proved to be a better fit (0.93 > R^2^ < 0.99; Figure 3) over the thermal time to anthesis than the logistic model used to track the flag leaf senescence (0.82 > R^2^ < 0.99).

### 3.2. Stay-Green Expression and Their Relationship with Yield and Yield Components

The stay-green genotype is characterized by a delayed senescence phenotype that allows plants to retain high leaf chlorophyll contents and, consequently, to maintain their leaves photo-synthetically to improve the grain-filling process [14]. Hence, the stay-green trait has been considered an important selection criterion for increasing crop production and stress tolerance, and is expressed as different combinations of delayed onset and a reduced rate of senescence across the genotypes [34]. Tomas and Howarth (2000) [34] describe five types of stay-green phenotype, which are classified into two large categories, called cosmetic and functional stay-green. Cosmetic phenotypes present an alteration in the catabolism of chlorophyll, while functional stay-green phenotypes are characterized by an altered timing of leaf senescence that leads to the maintenance of photosynthetic capacity in green tissues.

The results presented here indicated that leaf and senescence traits associated with stay-green expression were correlated with grain yield (GY) and yield components (Table 4), including thousand-kernel weight (TKW), harvest index (HI), shoot dry matter (biomass), number of kernels per spike (KS) and spikes per square meter (SM2). Except for SM2 and KS, this is in agreement with previous results reported in different crop plant species, including oats [33], sorghum [35], maize [31], durum wheat [36] and spring wheat [19].

Although SM2 is determined by the duration of the vegetative phase and does not overlap with the onset of senescence, the genotypes LE 2384 and LE 2367 coming from INIA-Uruguay (Table 6) showed a late flowering phenotype about 122 days to anthesis and the highest SM2 (Table 5), contrary to genotype QUP2616 from INIA-Chile, which recorded the earliest anthesis about 115 days from sowing and the lowest SM2.

While a significant correlation between stay-green and agronomical traits was observed, the Pearson correlation coefficients between these traits were moderate in the glasshouse experiment (Table 4). Moreover, leaf senescence traits estimated under field conditions showed a higher association with GY (r = 0.54–0.62) and biomass (r = 0.61–0.72), compared to the glasshouse experiment. Furthermore, the canopy senescence traits were highly correlated with all the agronomical traits, including GY (r = 0.43–0.65), biomass (r = 0.44–0.63), SM2 (r = 0.42–0.61) and KS (r = 0.48–0.55). Our results indicate that single measurements of chlorophyll content and/or NDVI taken during the grain filling period were strongly correlated with GY and yield components (Table 4). This agrees with previous results in which a single measurement of NDVI could be sufficient to select stay-green genotypes with higher yield in large populations in the field, under both well-watered and water-limited conditions [32,37]. However, unexpected negative associations between stay-green and GY have been also reported in rice [38] and wheat [15]. Additionally, a delayed senescence phenotype can also be associated with a decrease in wheat grain nutrient content due to limited remobilization of mineral nutrients from photosynthetic tissues [9]. Therefore, to ensure gains in yield potential through delayed leaf senescence, this trait should be combined with an increase in the grain filling capacity.

Genotypic variability for agronomical and physiological traits under different water stress scenarios and full irrigation conditions is of great interest for breeders because selected genotypes with favorable traits can be used as parents in future crosses for breeding programs. In the case of modern maize varieties released between 1930 and 2000, yield genetic gains were positively associated with the stay-green phenotype (reviewed by Thomas and Ougham (2014) [14]). In wheat, several studies have reported positive correlations between GY and delayed senescence under stress conditions, particularly under scenarios of progressive drought [32] and heat stress [23,25]. Thus, the assisted selection of stay-green traits or a delay in leaf senescence, together with the selection of agronomic traits associated with greater yield potential, and the use of molecular markers associated with these physiological and agronomical traits, will play an important role in the genetic progress towards more stable yields and the adaptation of crops to climate change.

The results presented here indicated that under the WL regime, the canopy senescence traits showed higher correlations with GY and yield components than the stay-green traits estimated at leaf level (Supplementary Material Table S1). Interestingly, in response to the WL regime, the genotype QUP2418 showed a decreased canopy senescence rate and delayed onset of leaf senescence (Table 1 and Table 2), which was associated with higher GY and TKW among genotypes (Table 5).

Overall, the present study’s results highlighted the contributions of the stay-green expression to yield and yield components under contrasting water regimes. Furthermore, the use of proximal sensing and high throughput phenotyping methodologies offer the potential for use as selection tools because of their high association with GY and yield components and remains a promising tool for breeding programs, together with the selection of secondary traits to further improve yield under stress.

## 4. Materials and Methods

### 4.1. Plant Material and Growing Conditions

Two experiments were conducted in glasshouse and field conditions with a set of fourteen contrasting spring bread wheat (*Triticum aestivum* L.) genotypes, listed in Table 6. The genotypes were selected according to the yield tolerance index from a previous study [5,39]. Cultivars PANTERA-INIA and PANDORA-INIA feature a similar genetic background, but PANTERA-INIA is a Clearfield^®^ cultivar with resistance to the herbicide imidazolinone following the introduction of the Ser-Asn627 mutation into two acetolactate synthase (ALS) genes (*imi1* and *imi2*), located in wheat on chromosomes 6B and 6D, respectively, into cv. PANDORA-INIA [40].

### 4.2. Glasshouse Experiment

In 2015, a glasshouse experiment was conducted at the Plant Breeding and Phenomic Center, Universidad de Talca, Talca, Chile (35°24′19″ S, 71°37′59″ W). The glasshouse featured natural lighting and a heating system; the average temperature of the growing period was 20 °C and the relative humidity 48% (Supplementary Material Figure S1). On 3 July 2015, ten seeds of each genotype were sown in 7.5 L circular pots filled with a 1:1:1 substrate mixture of organic soil (Anasac, Santiago, Chile), perlite, and river sand, representing a total dry weight per pot of 4.9 kg. After the emergence of the second leaf, the seedlings were thinned to five per pot. Two irrigation treatments were established from fully expanded flag leaf (Zadoks stage Z41) [41]: 30% (water-limited) and 75% (well-watered) of field capacity of the substrate mixture. Before the establishment of treatments, pots were weighed and watered to 75% of field capacity and fertilized with Hoagland nutritive solution (Caisson Lab, Smithfield, UT, USA). Soil water content was monitored by an automatic EC-5 sensor (Decagon Devices Inc., Pullman, WA, USA) connected to an EM-50 data logger (Decagon Devices Inc., Pullman, WA, USA; Supplementary Material Figure S2). The experiment was conducted in a randomized block design with four replications.

### 4.3. Field Experiment

The field experiment was set up during the 2017 season in Santa Rosa (36°32′ S, 71°55′ W), in the Mediterranean region of Chile. The genotypes were grown under well-watered (WW) and water-limited (WL) conditions. The cumulative rainfall during the experiment was 486 mm. Daily weather records (Supplementary Material Figure S3) were obtained from a nearby station of the Instituto de Investigaciones Agropecuarias (INIA), Chillán, Chile.

The experimental design was a randomized complete block design with four replicates. Plots consisted of five rows of 2 m in length and 0.2 m distance between rows. The sowing rate was 20 g m^−2^ and the sowing date was 18 July. Plots were fertilized with 260 kg ha^−1^ of ammonium phosphate (46% P_2_O_5_ and 18% N), 90 kg ha^−1^ of potassium chloride (60% K_2_O), 200 kg ha^−1^ of sulpomag (22% K_2_O, 18% MgO and 22% S), 10 kg ha^−1^ of boronatrocalcite (11% B) and 3 kg ha^−1^ of zinc sulfate (35% Zn). Fertilizers were incorporated with a cultivator before sowing. During tilling, an extra 153 kg ha^−1^ of N was applied. Weeds were controlled with the application of Flufenacet + Flurtamone + Diflufenican (96 g a.i.) as pre-emergence controls and a further application of MCPA (525 g a.i.) + Metsulfuron-methyl (5 g a.i.) as post-emergents. Cultivars were disease-tolerant and no fungicide was used. Furrow irrigation was used for the WW condition: three irrigations of about 50 mm at flag leaf stage (Z37), heading (Z50), and middle grain filling (Z70). For the WL regime (rainfed condition), plots received the natural precipitation until heading, and after that, a plastic shelter was used to prevent rainfall during grain filling.

### 4.4. Anthesis Time and Thermal Time

In both experiments, the anthesis date was recorded through periodic observations, when approximately half of the spikes had already extruded anthers from the middle spikelets. Calendar dates of anthesis were then converted into accumulated thermal time (degree days, °Cd). Daily thermal time was calculated as the average of the maximum and minimum air temperature considering a base temperature of 0 °C.

### 4.5. Leaf Senescence

Leaf senescence was assessed by the non-destructive measurement of leaf chlorophyll content index (Chl) using a portable chlorophyll meter (Dualex Scientific, Force A, Orzay, France). In the glasshouse experiment, five leaves per pot were measured from flag leaf emergence onwards. In the field experiment, measurements were taken on six leaves, three points along each leaf, starting in the initial stages of grain filling. All measurements were taken on healthy, clean flag leaves in both experiments.

Data of Chl was fitted over the accumulated thermal time after anthesis (x) using a logistic function, with a model similar to that described by Xie et al. (2014) [13]:

(1) $$Chl=\frac{Chl_{\max}}{1+{\left(\frac{x}{t_{50}}\right)}^{rate}}$$

where *t*_50_ is the thermal time to 50% senescence, the *rate* is the rate of leaf senescence and *Chl*_max_ corresponds to the maximum chlorophyll accumulated.

**Figure 6:**
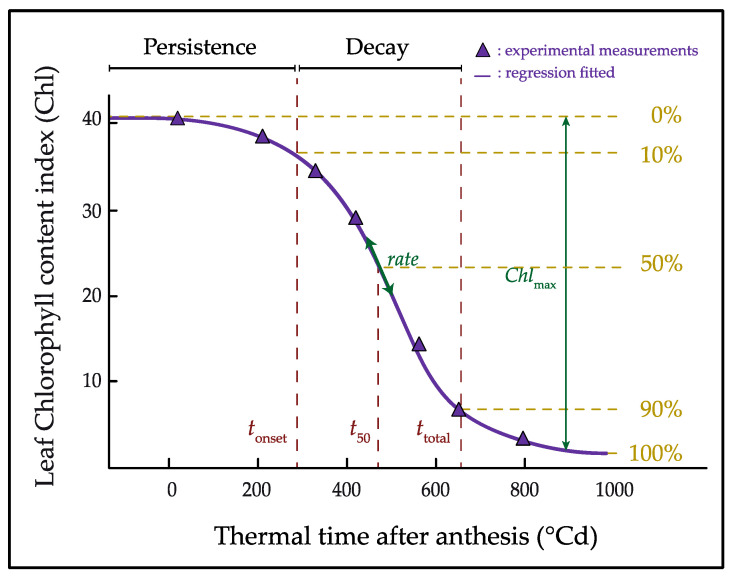
Graphical representation of the logistic function fitted for flag leaf senescence and the estimated stay-green traits. Total duration of leaf senescence (*t*_total_) is defined as the period from anthesis to the time when 90% of chlorophyll content has been lost (90% senescence). The *t*_total_ is divided in two phases: the chlorophyll persistence phase, from anthesis to the time when reach 10% senescence (*t*_onset_), and the decay phase (*decay*) from *t*_onset_ to *t*_total_. *Rate* indicates the rate of chlorophyll loss, *Chl*_max_ correspond to the maximum chlorophyll content, and *t*_50_ indicate the time when reach the maximum senescence rate.

The curve fitting was used to estimate the following stay-green traits for each pot or plot: (i) the chlorophyll persistence phase, defined as the period between anthesis and the time when reaching 10% senescence (*t*_onset_); (ii) the decay phase (*decay*) from *t*_onset_ to *t*_total_; (iii) the total duration of flag leaf senescence (*t*_total_), i.e., the period from anthesis to 90% loss of *Chl*_max_; and (iv) the area under Chl curve (*Area*_Chl_) was calculated from anthesis to 1000 °Cd, as a measure of the total flag leaf greenness of a genotype (Figure 6). Curve fittings were performed with SigmaPlot 10.0 and the area under the Chl curve was calculated with the AREA.XFM transform.

### 4.6. Canopy Senescence

Canopy senescence was assessed by measuring the Normalized Difference Vegetation Index (NDVI) of each plot with a hand-held crop sensor (GreenSeeker, Trimble, CA, USA). The distance between the GreenSeeker and the plot was kept constant at around 60 cm, measuring only the central row to avoid pointing border rows. A total of eight measurements were taken during grain filling, starting in the initial stages and ending when all plots attained physiological maturity.

**Figure 7:**
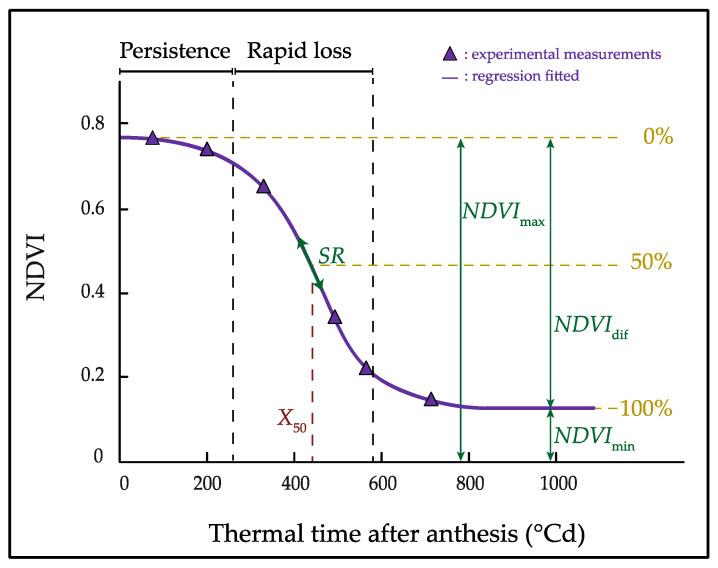
Graphical representation of the sigmoid function fitted for canopy senescence and the estimated stay-green traits. *SR* indicate the rate of canopy senescence, *NDVI*_max_ correspond to the maximum canopy greenness (*NDVI*_max_ = *NDVI*_dif_ + *NDVI*_min_), and *X*_50_ indicate the time when reach the maximum senescence rate (50% senescence).

NDVI data were then fitted over the accumulated thermal time (x) after anthesis using a sigmoid function with a model similar to that described by Christopher et al. (2014) [37]:

(2) $$NDVI=NDVI_{\min}+\frac{NDVI_{\mathrm{dif}}}{\left(1+e^{\left(-\frac{\left(x-x_{50}\right)}{SR}\right)}\right)}$$

where *SR* is the rate of canopy senescence, *NDVI*_dif_ corresponds to the difference in NDVI between the maximum (*NDVI*_max_) and minimum (*NDVI*_min_) value, and *X*_50_ is the thermal time to 50% senescence. From the fit of NDVI versus thermal time (Figure 7), the following stay-green trait was estimated for each plot: the area under NDVI curve (*Area*_NDVI_), calculated between anthesis to 1000 °Cd, as a measure of the canopy greenness of a genotype. Curve fittings were performed with SigmaPlot 10.0, and the area under the NDVI curve was calculated with AREA.XFM transform.

### 4.7. Agronomical Traits

In the glasshouse experiment, plants were harvested at maturity and dried in a fan-forced oven at 60 °C for 48 h. The spikes were counted and threshed manually. The following traits were evaluated at maturity: grain yield (GY), number of kernels per spike (KS), thousand-kernel weight (TKW), and harvest index (HI). In the field experiment, 1 m of an inside row was harvested at maturity and dried in the oven to determine HI. KS and TKW were determined from 25 spikes taken at random from the inside row. GY was assessed by harvesting the whole plot.

### 4.8. Statistical Analysis

Analysis of variance (ANOVA) for each phenotypic trait was applied to assess the effect of genotype (G), water regime (W: WL and WW), and their interaction. Pearson correlations between different traits were computed using the average values across replicates. Phenotypic data were transformed to improve the normality of trait distribution when necessary. All the statistics and graphics were performed using the Statistical Software R version 3.3.3.

## 5. Conclusions

The present study highlighted the contributions of the stay-green expression to the grain yield (GY) and yield components under contrasting water regimes. Furthermore, the proximal sensing and high-throughput phenotyping methodologies offer potential as selection tools in breeding programs because of their high association with GY and yield components. In the present study, significant differences in the dynamics of leaf and canopy senescence (stay-green traits) were found between well-watered (WW) and water-limited (WL) regimes and among cultivars. Our results also revealed a positive correlation between canopy stay-green traits and GY under both water regimes. QUP2418 exhibited better agronomic performance under the WW and WL regimes and was consistent across the field and glasshouse experiments. Therefore, the advanced-line QUP2418 with high grain yield and thousand-kernel weight and a decreased canopy senescence rate and delayed onset of leaf senescence can be an interesting source to be utilized in physiological breeding for the future genetic improvement of spring bread wheat under terminal water stress.

## Figures and Tables

**Table 1:** Mean values of *Chl*_max_ (dualex units), *rate* (dualex units °Cd^−1^), *t*_50_ (°Cd), *t*_onset_ (°Cd), *t*_total_ (°Cd), decay (°Cd) and *Area*_chl_ (Mdualex units °Cd^−1^) for a set of contrasting spring bread wheat genotypes grown under water-limited (WL) and well-watered (WW) regimes in glasshouse and field conditions. Significance levels for the analysis of variance (ANOVA) are also shown.

	**Glasshouse Experiment**													
	***Chl*** **_max_**		***Rate***		***t*** **_50_**		***t*** **_onset_**		***t*** **_total_**		***Decay***		***Area*** **_chl_**	
**Genotype**	**WW**	**WL**	**WW**	**WL**	**WW**	**WL**	**WW**	**WL**	**WW**	**WL**	**WW**	**WL**	**WW**	**WL**
Fontagro 8	44	45	5.6	6.2	638	682	430	477	952	976	522	500	28.9	31.0
Fontagro 92	49	48	5.8	6.6	784	683	533	490	1159	954	627	464	38.3	33.3
Fontagro 98	48	47	6.0	7.5	696	690	481	507	1010	945	529	438	34.1	33.0
LE 2367	43	43	4.9	7.5	818	801	512	575	1319	1131	807	556	33.8	34.2
LE 2384	45	46	8.0	7.8	772	824	580	616	1032	1105	452	488	34.7	37.1
Pandora-INIA	49	48	4.9	6.1	711	771	456	528	1110	1132	654	603	34.8	36.7
Pantera-INIA	47	47	4.7	6.0	697	727	434	496	1120	1072	686	575	32.8	34.2
QUP2405	46	47	4.5	5.6	774	812	476	542	1262	1219	787	677	34.8	36.6
QUP2418	45	44	6.3	6.8	803	738	559	534	1163	1020	604	487	35.6	32.8
QUP2474	44	45	4.7	5.6	661	702	414	474	1061	1041	647	567	29.8	31.9
QUP2529	43	43	6.2	7.5	667	717	465	522	963	990	498	468	29.3	31.0
QUP2546	nc	nc	nc	nc	nc	nc	nc	nc	nc	nc	nc	nc	nc	nc
QUP2569	43	44	7.6	8.2	739	738	554	558	986	982	432	424	32.0	32.9
QUP2616	50	52	6.7	7.7	709	711	505	532	999	952	494	420	35.9	37.2
Treatment														
WW	46		5.8		728		492		1087		595		33	
WL	46		6.9		738		527		1040		513		34	
ANOVA														
Genotype (G)	***		***		***		***		***		***		***	
Water regime (W)	ns		***		ns		**		*		***		ns	
G × W	ns		ns		ns		ns		ns		ns		ns	
	**Field Experiment**													
	***Chl*** **_max_**		***Rate***		***t*** **_50_**		***t*** **_onset_**		***t*** **_total_**		***Decay***		***Area*** **_chl_**	
**Genotype**	**WW**	**WL**	**WW**	**WL**	**WW**	**WL**	**WW**	**WL**	**WW**	**WL**	**WW**	**WL**	**WW**	**WL**
Fontagro 8	38	36	6.7	10.6	432	336	310	260	604	441	294	181	17.1	12.3
Fontagro 92	nc	nc	nc	nc	nc	nc	nc	nc	nc	nc	nc	nc	nc	nc
Fontagro 98	39	38	8.3	6.9	454	420	345	306	602	577	257	270	18.0	16.7
LE 2367	33	33	7.5	6.4	422	378	314	268	570	536	256	268	14.4	12.9
LE 2384	34	34	8.6	6.9	414	375	318	272	541	517	224	245	14.3	13.2
Pandora-INIA	41	41	8.6	6.8	411	353	312	250	545	503	233	252	17.3	14.9
Pantera-INIA	39	38	8.0	6.9	402	360	301	262	542	496	241	233	16.2	14.2
QUP2405	38	38	9.1	7.7	467	389	363	288	605	531	242	243	18.4	15.2
QUP2418	34	36	8.5	8.2	502	444	382	331	665	603	283	272	17.7	16.4
QUP2474	36	34	7.2	7.6	429	358	314	262	587	494	273	232	13.9	12.7
QUP2529	37	35	9.0	6.7	453	382	348	274	592	533	243	259	17.3	14.0
QUP2546	42	39	8.3	8.3	433	424	331	323	566	560	234	237	17.0	15.2
QUP2569	38	38	8.5	6.9	392	396	302	285	509	553	207	268	15.1	15.4
QUP2616	43	42	9.4	7.6	464	471	367	353	587	628	220	274	20.3	18.7
Treatment														
WW	38		8.3		437		331		578		247		17	
WL	37		7.5		391		287		536		249		15	
ANOVA														
Genotype (G)	***		ns		**		ns		**		ns		*	
Water regime (W)	ns		ns		***		***		***		ns		***	
G × W	ns		ns		ns		ns		ns		ns		ns	

*, ** and *** indicate significance difference at 0.05, 0.01, and 0.001 level, respectively. ns: non-significant. nc: not calculated. *Chl*_max_, maximum chlorophyll accumulated; *rate*, indicator of rate of senescence; *t*_onset_, thermal time from anthesis to 10% senescence; *t*_50_, thermal time from anthesis to loss 50% of *Chl*_max_; *t*_total_, thermal time from anthesis to 90% senescence; *decay*, thermal time from 10 to 90% senescence; *Area*_chl_, thermal time from anthesis to 1000 °Cd.

**Table 2:** Mean values of *NDVI*_dif_ (NDVI units), *SR* (NDVI units °Cd^−1^), *X*_50_ (°Cd), *NDVI*_max_ (NDVI units), and *Area*_NDVI_ (°Cd) for a set of contrasting spring bread wheat genotypes grown under water-limited (WL) and well-watered (WW) regimes in field conditions. Significance levels for the analysis of variance (ANOVA) are also shown.

	*NDVI*_dif_		*SR*		*X*_50_		*NDVI*_max_		*Area*_NDVI_	
Genotype	WW	WL	WW	WL	WW	WL	WW	WL	WW	WL
Fontagro 8	0.50	0.49	−87.1	−89.6	429	345	0.65	0.64	365	320
Fontagro 92	0.51	0.47	−96.7	−72.6	485	429	0.66	0.63	396	366
Fontagro 98	0.52	0.46	−97.3	−70.6	480	462	0.67	0.62	398	375
LE 2367	0.59	0.55	−105.9	−103.3	489	437	0.75	0.72	447	407
LE 2384	0.56	0.56	−107.2	−103.8	453	372	0.71	0.71	404	360
Pandora-INIA	0.52	0.50	−126.2	−91.7	446	357	0.67	0.65	385	330
Pantera-INIA	0.56	0.53	−128.6	−98.9	413	378	0.71	0.68	384	353
QUP2405	0.56	0.49	−151.1	−111.1	391	375	0.71	0.64	372	334
QUP2418	0.55	0.51	−140.7	−102.7	480	424	0.70	0.66	414	366
QUP2474	0.51	0.46	−77.8	−63.4	466	374	0.66	0.64	389	347
QUP2529	0.55	0.52	−96.5	−79.4	432	373	0.70	0.67	386	344
QUP2546	0.52	0.49	−118.6	−97.6	466	422	0.67	0.65	391	365
QUP2569	0.56	0.54	−89.2	−85.9	459	403	0.71	0.69	409	375
QUP2616	0.48	0.48	−107.2	−95.6	467	435	0.63	0.65	371	377
Treatment										
WW	0.53		−109		454		0.68		394	
WL	0.50		−90		399		0.66		358	
ANOVA										
Genotype (G)	***		***		***		***		***	
Water regime (W)	***		***		***		***		***	
G × W	ns		ns		ns		ns		ns	

*** indicate significance difference at 0.001 level, respectively. ns, non-significant. *NDVI*_dif_ corresponds to the difference in NDVI between the maximum (*NDVI*_max_) and minimum (*NDVI*_min_) value; *SR*, indicator of the rate of canopy senescence; *X*_50_, thermal time from anthesis to loss 50% of *NDVI*_dif_; *Area*_NDVI_, thermal time from anthesis to 1000 °Cd.

**Table 3:** Mean values of grain yield (GY; g per plant), number of kernels per spike (KS), thousand-kernel weight (TKW; g) and harvest index (HI) for fourteen genotypes grown under water-limited (WL) and well-watered (WW) regimes in glasshouse conditions. Significance levels for the analysis of variance (ANOVA) are also shown.

	GY		KS		TKW		HI	
Genotypes	WW	WL	WW	WL	WW	WL	WW	WL
Fontagro 8	16.31	13.18	50.41	48.34	50.97	57.04	0.57	0.57
Fontagro 92	19.84	14.37	52.60	46.24	52.15	56.04	0.57	0.57
Fontagro 98	15.33	11.29	43.81	46.04	60.03	57.09	0.47	0.46
LE 2384	18.81	14.13	51.13	49.49	45.43	49.05	0.51	0.53
LE 2367	18.92	14.31	51.47	51.65	45.85	46.34	0.52	0.55
Pandora-INIA	17.96	13.42	59.65	50.07	54.52	57.84	0.55	0.55
Pantera-INIA	15.37	13.59	54.40	51.68	54.55	56.09	0.55	0.57
QUP2418	20.44	13.83	48.93	46.60	60.64	61.07	0.56	0.54
QUP2405	15.49	13.15	54.88	59.85	45.90	51.46	0.55	0.58
QUP2474	17.47	13.94	51.35	47.13	53.65	52.04	0.53	0.54
QUP2529	16.60	13.03	53.35	48.60	48.01	50.05	0.53	0.55
QUP2546	13.97	10.10	46.65	44.57	44.58	49.07	0.38	0.37
QUP2569	16.25	12.80	52.37	55.94	52.99	53.30	0.47	0.48
QUP2616	15.61	11.32	52.93	48.20	55.60	57.15	0.54	0.56
Treatment								
WW	17.02		51.72		51.74		0.52	
WL	13.06		49.66		53.77		0.53	
ANOVA								
Genotype (G)	***		**		***		***	
Water regime (W)	***		*		***		ns	
G × W	ns		ns		ns		ns	

*, ** and *** indicate significance difference at 0.05, 0.01, and 0.001 level, respectively. ns, non-significant.

**Table 4:** Correlations between agronomical and stay-green traits in fourteen selected bread wheat genotypes evaluated in glasshouse and field experiments, grown under well-watered and water-limited regimes.

Glasshouse Experiment					Field Experiment				
Stay-Green Traits	GY (g Plant^−1^)	TKW (g)	HI	KS	Stay-Green Traits	GY (*t* ha^−1^)	Biomass (g)	SM2	KS
Leaf senescence:					Leaf senescence:				
*Chl*_max_	−0.16	0.44 *	0.20	0.00	*Chl*_max_	−0.03	0.02	−0.28	−0.10
*rate*	−0.38	0.02	−0.38	−0.30	*rate*	0.20	0.25	0.09	0.03
*t*_50_	0.22	−0.31	0.07	0.25	*t*_50_	0.60 **	0.70 ***	0.49 *	−0.28
*t*_onset_	−0.08	−0.14	−0.17	−0.05	*t*_onset_	0.62 **	0.72 ***	0.49 *	−0.27
*t*_total_	0.42 *	−0.36	0.27	0.44 *	*t*_total_	0.54 **	0.61 **	0.44 *	−0.26
*decay*	0.44 *	−0.28	0.34	0.44 *	*decay*	0.18	0.18	0.16	−0.12
*Area*_chl_	0.05	0.10	0.18	0.16	*Area*_chl_	0.43 *	0.51 *	0.16	−0.30
Chla	−0.10	0.45 *	0.26	−0.02	Chla	0.31	0.35	0.09	−0.31
Chlgf	−0.24	0.24	0.00	−0.01	Chlgf	0.71 ***	0.66 ***	0.63 **	−0.53 **
Canopy senescence:					Canopy senescence:				
*NDVI*_dif_					*NDVI*_dif_	0.65 ***	0.60 ***	0.61 ***	−0.55 **
*SR*					*SR*	−0.55 **	−0.44 *	−0.33	0.51 **
*X*_50_					*X*_50_	0.43 *	0.54 **	0.42 *	−0.14
*NDVI*_max_					*NDVI*_max_	0.57 **	0.53 **	0.58 **	−0.48 **
*Area*_NDVI_					*Area*_NDVI_	0.56 **	0.63 ***	0.58 **	−0.29
NDVIa					NDVIa	0.65 ***	0.68 ***	0.65 ***	−0.48 *
NDVIgf					NDVIgf	0.84 ***	0.83 ***	0.76 ***	−0.66 ***

Coefficients of significance correlations are in bold character. *, ** and *** indicate significance difference at 0.05, 0.01, and 0.001 level, respectively. na, not-available. GY, grain yield; KS, number of kernel per spike; HI, harvest index; SM2, Spikes per square meter; *Chl*_max_, maximum chlorophyll accumulated; *rate*, indicator of rate of senescence; *t*_onset_, thermal time from anthesis to 10% senescence; *t*_50_, thermal time from anthesis to loss 50% of *Chl*_max_; Chla and Chlgf, Chl content values recorded at anthesis and grain filling, respectively; *t*_total_, thermal time from anthesis to 90% senescence; *decay*, thermal time from 10 to 90% senescence. *NDVI*_dif_; correspond to the difference in NDVI between the maximum (*NDVI*_max_) and minimum (*NDVI*_min_) value; *SR*, indicator of the rate of canopy senescence; *X_50_*, thermal time from anthesis to loss 50% of *NDVI*_dif_; NDVIa and NDVIgf, NDVI values recorded at anthesis and grain filling, respectively.

**Table 5:** Mean values of grain yield (GY; *t* ha^−1^), number of kernels per spike (KS), thousand-kernel weight (TKW; g), harvest index (HI) and number of spikes per square meter (SM2) for fourteen genotypes grown under water-limited (WL) and well-watered (WW) regimes in field conditions. Significance levels for the analysis of variance (ANOVA) are also shown.

	GY		KS		TKW		HI		SM2	
Genotypes	WW	WL	WW	WL	WW	WL	WW	WL	WW	WL
Fontagro 8	10.31	5.73	23.90	34.51	48.35	48.92	0.47	0.43	418.75	336.25
Fontagro 92	7.99	5.27	28.53	39.32	48.19	49.88	0.49	0.43	408.75	331.25
Fontagro 98	8.49	5.74	27.04	32.26	48.73	48.43	0.42	0.41	380.00	353.75
LE 2367	9.46	7.06	26.93	27.58	45.86	45.46	0.46	0.48	470.00	403.75
LE 2384	10.95	7.80	23.59	26.69	41.25	41.00	0.48	0.53	432.50	440.00
Pandora-INIA	9.42	6.69	19.24	28.24	46.94	56.61	0.50	0.48	378.75	278.75
Pantera-INIA	10.34	8.17	22.85	22.62	50.09	48.71	0.50	0.50	367.50	345.00
QUP2405	9.37	6.60	21.53	30.97	40.87	44.67	0.49	0.49	416.25	352.50
QUP2418	10.71	7.85	25.90	29.79	55.15	54.50	0.47	0.47	388.75	326.25
QUP2474	9.92	6.86	23.39	27.78	47.17	48.19	0.47	0.45	422.50	311.25
QUP2529	9.48	7.17	25.99	27.17	45.57	41.96	0.46	0.46	445.00	358.75
QUP2546	8.88	7.04	28.68	31.49	49.01	49.03	0.43	0.42	407.50	390.00
QUP2569	8.87	6.47	27.37	31.97	49.55	50.41	0.43	0.41	352.50	332.50
QUP2616	8.11	5.60	26.31	32.16	48.19	50.29	0.48	0.45	347.50	268.75
Treatment										
WW	9.45		25.09		47.49		0.47		402.59	
WL	6.72		30.18		48.43		0.46		344.91	
ANOVA										
Genotype (G)	***		***		***		***		*	
Water regime (W)	***		***		ns		**		***	
G × W	*		**		*		ns		ns	

*, ** and *** indicate significance difference at 0.05, 0.01, and 0.001 level, respectively. ns, non-significant.

**Table 6:** The 14 selected wheat genotypes and their level of tolerance to water stress, according to the yield tolerance index (YTI) determined in two Mediterranean environments.

Genotype	Origin	YTI *	Tolerance to Stress
QUP2418	INIA-Chile	0.67	Tolerant
QUP2546	INIA-Chile	0.56	Tolerant
Fontagro 8	INIA-Chile	0.52	Tolerant
LE 2367	INIA-Uruguay	0.47	Tolerant
QUP2529	INIA-Chile	0.44	Tolerant
QUP2474	INIA-Chile	0.44	Tolerant
Pantera	INIA-Chile	0.38	Tolerant
Fontagro 92	CIMMYT-Mexico	0.37	Tolerant
QUP2405	INIA-Chile	0.36	Tolerant
QUP2616	INIA-Chile	0.33	Susceptible
LE 2384	INIA-Uruguay	0.31	Susceptible
Pandora	INIA-Chile	0.26	Susceptible
QUP2569	INIA-Chile	0.21	Susceptible
Fontagro 98	CIMMYT-Mexico	0.15	Susceptible

*: Higher values indicate a better performance of the genotype under rainfed areas [5].

## Data Availability

The data are available from the corresponding author upon reasonable request.

## Supplementary Materials

The following are available online at https://www.mdpi.com/article/10.3390/plants11030292/s1, Figure S1: Daily temperature and relative humidity during the growing period in the glasshouse trial; Figure S2: Volumetric water content during the growing period in the glasshouse trial; Figure S3: Daily weather records during the growing period in the field trial; Table S1: Correlations between agronomical and stay-green traits in a set of spring bread wheat genotypes grown under water-limited (WL) and well-watered (WW) regimes.
