# Supplementary material for: Leaf and Canopy Traits Associated with Stay-Green Expression Are Closely Related to Yield Components of Wheat Genotypes with Contrasting Tolerance to Water Stress
Source: Plants (Basel). 2022 Jan 22;11(3):292. doi: 10.3390/plants11030292 (PMC8838353; doi:10.3390/plants11030292)
Supplement: Supplementary file 1 [file plants-11-00292-s001.zip › plants-1543474-supplementary.pdf]

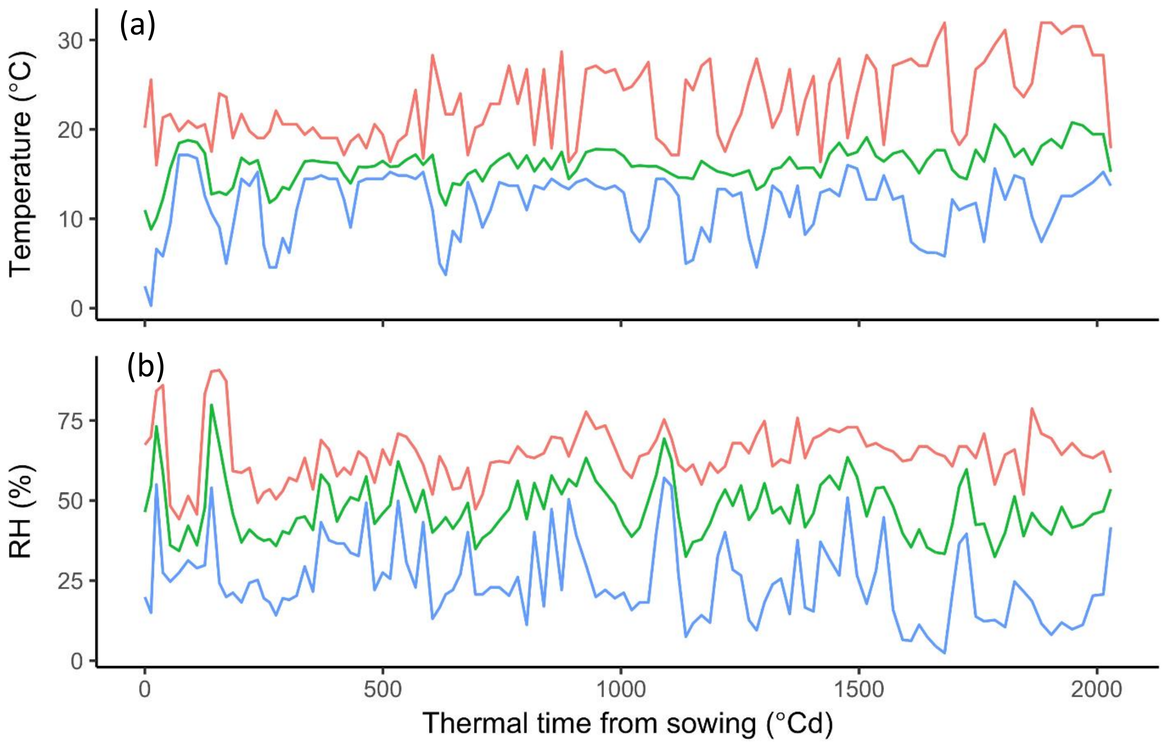

Figure S1. Temperature and relative humidity (RH) during the growing season of wheat genotypes in the glasshouse experiment. Graphs show the maximum (red line), minimum (blue line), and mean (green line) of temperature (top graph), and RH (bottom panel).

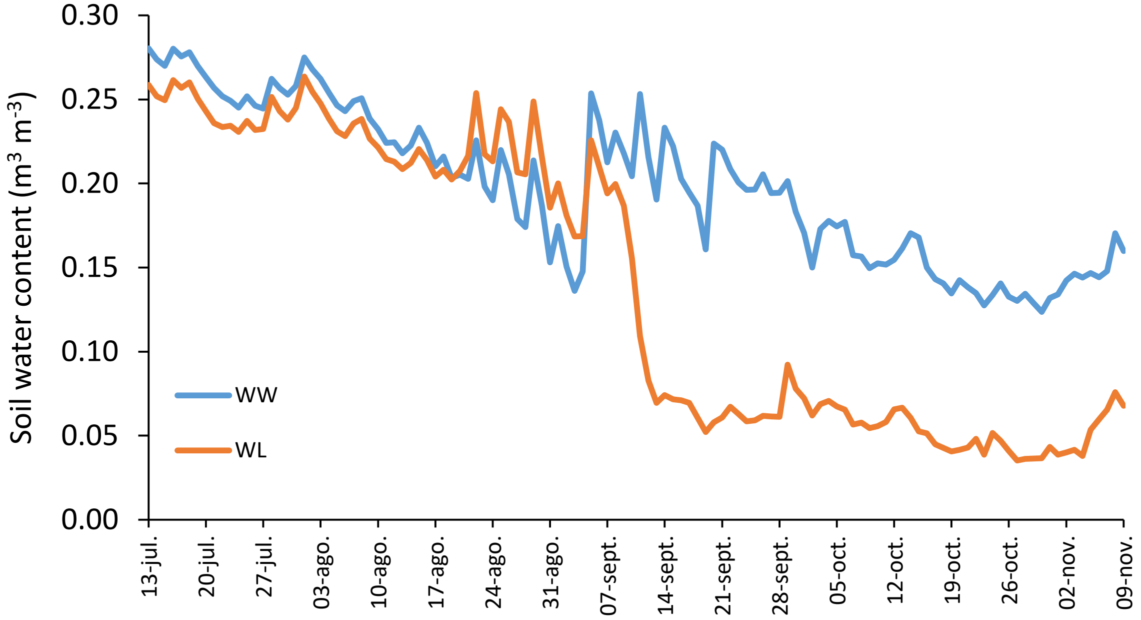

Figure S2. Volumetric water content from seedling emergence to physiological maturity in the glasshouse experiment. Two irrigation treatments were established from flag leaf fully expanded (Z41): 30% (water-limited; WL) and 75% (well-watered; WW) of field capacity.

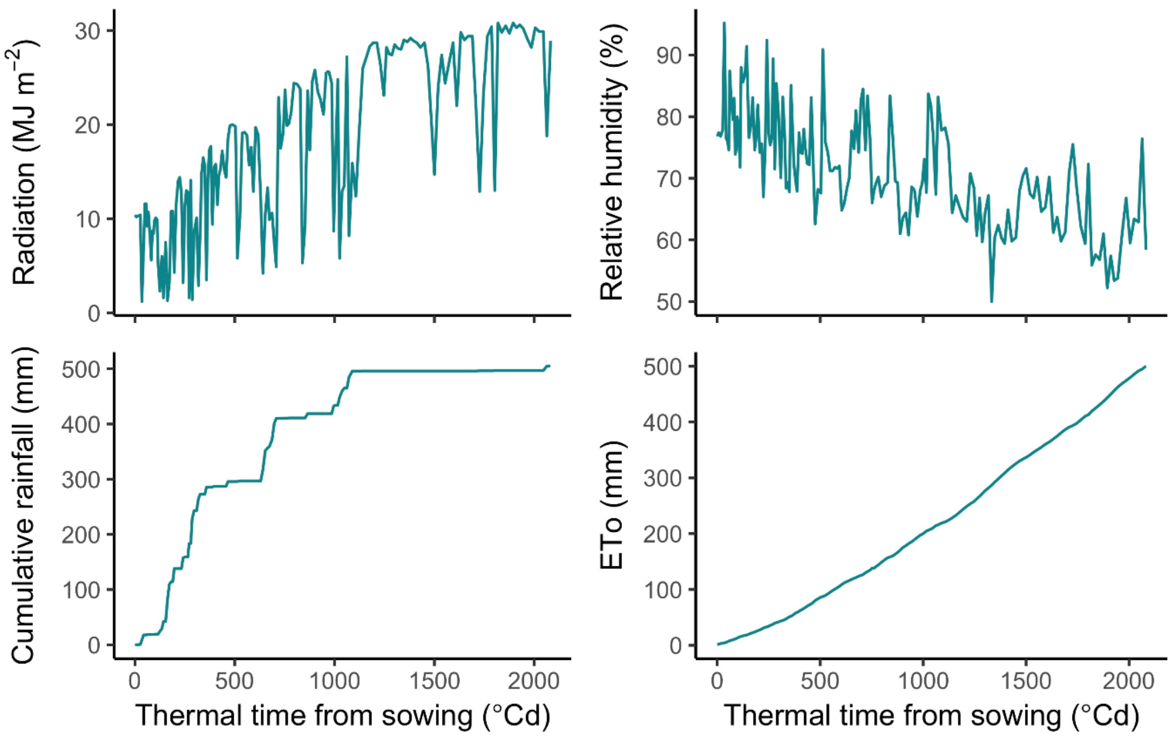

Figure S3. Daily solar radiation, relative humidity (RH), cumulative rainfall, and reference evapotranspiration (ETo) during the growing season of wheat genotypes in Santa Rosa (field experiment).

Table supplementary S1. Correlations between agronomical and stay-green traits genotypes in a set of spring wheat genotypes grown under water-limited (WL) and well-watered (WW) regimes in field conditions.

	WW					WL				
Stay-green traits	GY (t ha^{-1})	Biomass (g)	SM2	KS	HI	GY (t ha^{-1})	Biomass (g)	SM2	KS	HI
Leaf senescence:										
Chl_{max}	−0.53	**−0.63 ***	−0.51	−0.18	−0.08	−0.17	0.03	−0.45	0.17	−0.28
rate	−0.20	−0.31	−0.19	−0.08	0.04	−0.36	−0.20	−0.16	**0.60 ***	−0.31
t_{50}	0.10	0.22	0.18	0.13	−0.08	0.14	0.52	0.19	0.25	−0.33
t_{onset}	0.00	0.12	0.11	0.15	−0.12	0.07	0.49	0.18	0.39	−0.40
t_{total}	0.21	0.31	0.24	0.07	−0.01	0.19	0.49	0.16	0.11	−0.24
decay	0.34	0.38	0.28	−0.02	0.09	0.24	0.33	0.08	−0.20	0.00
$Area_{Chl}$	−0.26	−0.32	−0.29	−0.1	−0.02	−0.47	0.25	−0.23	0.24	−0.34
Chla	−0.48	**−0.62 ***	−0.41	−0.21	−0.01	−0.00	0.15	−0.23	0.15	−0.14
Chlgf	0.13	−0.15	0.38	−0.23	0.36	0.57	0.47	0.41	−0.15	0.33
Canopy senescence:										
$NDVI_{dif}$	0.46	0.47	0.36	−0.11	0.00	**0.66 ***	0.46	**0.58 ***	**−0.57 ***	**0.57 ***
SR	−0.21	0.00	0.14	0.37	−0.36	−0.47	−0.19	−0.32	0.31	**−0.59 ***
X_{50}	−0.33	0.00	0.00	**0.65***	−0.43	−0.28	−0.06	0.05	0.33	−0.42
$NDVI_{max}$	0.44	0.46	0.39	−0.09	−0.00	**0.58 ***	0.41	**0.55 ***	**−0.54 ***	0.50
$Area_{NDVI}$	0.09	0.28	0.36	0.40	−0.32	0.00	0.11	0.29	0.03	−0.14
NDVIa	0.34	0.46	0.50	0.14	−0.19	**0.61 ***	**0.56 ***	**0.61 ***	**−0.57 ***	0.33
NDVIgf	0.31	0.51	**0.55 ***	0.19	−0.33	**0.71 ****	**0.68 ****	**0.63 ***	**−0.64 ***	0.34

Coefficients of significance correlations are in bold character. * and ** indicate significance difference at 0.05 and 0.01 level, respectively. GY, grain yield; KS, number of kernel per spike; HI, harvest index; SM2, Spikes per square meter; Chl_{max}, maximum chlorophyll accumulated; *rate*, indicator of rate of senescence; t_{onset}; thermal time from anthesis to 10% senescence; t_{50}; thermal time from anthesis to loss 50% of Chl_{max}; t_{total}, thermal time from anthesis to 90% senescence; *decay*, thermal time from 10 to 90% senescence. $NDVI_{dif}$; correspond to the difference in NDVI between the maximum ($NDVI_{max}$) and minimum ($NDVI_{min}$) value; *SR*, indicator of the rate of canopy senescence; X_{50}, thermal time from anthesis to loss 50% of $NDVI_{dif}$; $Area_{NDVI}$, thermal time from anthesis to 1000 °Cd.
